# Morphological responses to feeding in ticks *(Ixodes ricinus)*

**DOI:** 10.1186/s40851-018-0104-0

**Published:** 2018-08-04

**Authors:** J. Matthias Starck, Lisa Mehnert, Anja Biging, Juliana Bjarsch, Sandra Franz-Guess, Daniel Kleeberger, Marie Hörnig

**Affiliations:** 1 Functional Morphology Group, Department of Biology 2, University of Munich (LMU), Biocenter Martinsried, Großhadernerstr. 2, D-82152 Planegg-, Martinsried, Germany; 2 Zoological Institute and Museum, Cytology and Evolutionary Biology, Soldmannstr 23, D17487 Greifswald, Germany

**Keywords:** Cuticula, Epidermis, Midgut diverticula, Tracheae, Taenidia, Feeding, Volume change

## Abstract

**Background:**

Ticks can survive long periods without feeding but, when feeding, ingest large quantities of blood, resulting in a more than 100-fold increase of body volume. We study morphological adaptations to changes in opisthosoma volume during feeding in the castor bean tick, *Ixodes ricinus*. We aim to understand the functional morphological features that accommodate enormous changes in volume changes.

**Methods:**

Using light and electron microscopy, we compare the cuticle and epidermis of the alloscutum, the epithelium of the midgut diverticula, and the tracheae of adult female ticks when fasting, semi-engorged, and fully engorged.

**Results:**

Our results add to an existing body of knowledge that the area of the epidermis increases by cellular differentiation, cellular hypertrophy, and changes in the shape of epithelial cells from pseudostratified to single layered prismatic in semi-engorged ticks, and to thin squamous epithelium in fully engorged ticks. We did not find evidence for cell proliferation. The midgut diverticula accommodate the volume increase by cellular hypertrophy and changes in cell shape. In fully engorged ticks, the epithelial cells of the midgut diverticula are stretched to an extremely thin, squamous epithelium. Changes in size and shape (and cell divisions) contribute to the accommodation of volume changes. Tracheae do not increase in size, but extend in length, thus following the volume changes of the opisthosoma in feeding ticks to secure oxygen supply to the internal organs.

**Conclusions:**

Changes of epithelial tissue configuration in the epidermis and the midgut diverticula are described as important components of the morphological response to feeding in ticks. We provide evidence for a previously unknown mechanism hosted in the endocuticle of the tracheae that allows the tracheae of castor bean ticks to expand when the body volume increases and the distance between the respiratory spiracle and the oxygen demanding tissue enlarges. This is the first report of expandable tracheae in arthropods.

**Electronic supplementary material:**

The online version of this article (10.1186/s40851-018-0104-0) contains supplementary material, which is available to authorized users.

## Background

The castor bean tick, *Ixodes ricinus*, is a European species of hard-bodied ticks, which feeds on blood as a temporary ectoparasite of wild and domestic animals, as well as humans. *Ixodes ricinus* has a three-host life cycle, with larvae, nymphs and adults feeding on blood from their respective hosts. Ticks survive long periods of fasting and then ingest large amounts of blood in a few days. They are sexually dimorphic in behavior and morphology, with the males feeding as larvae and nymphs, but taking only occasional, small blood meals as adults. Females feed during all life stages. However, as adults they feed only one large blood meal, drop from their host when fully engorged, lay thousands of eggs, and die [1].

**Fig. 1:**
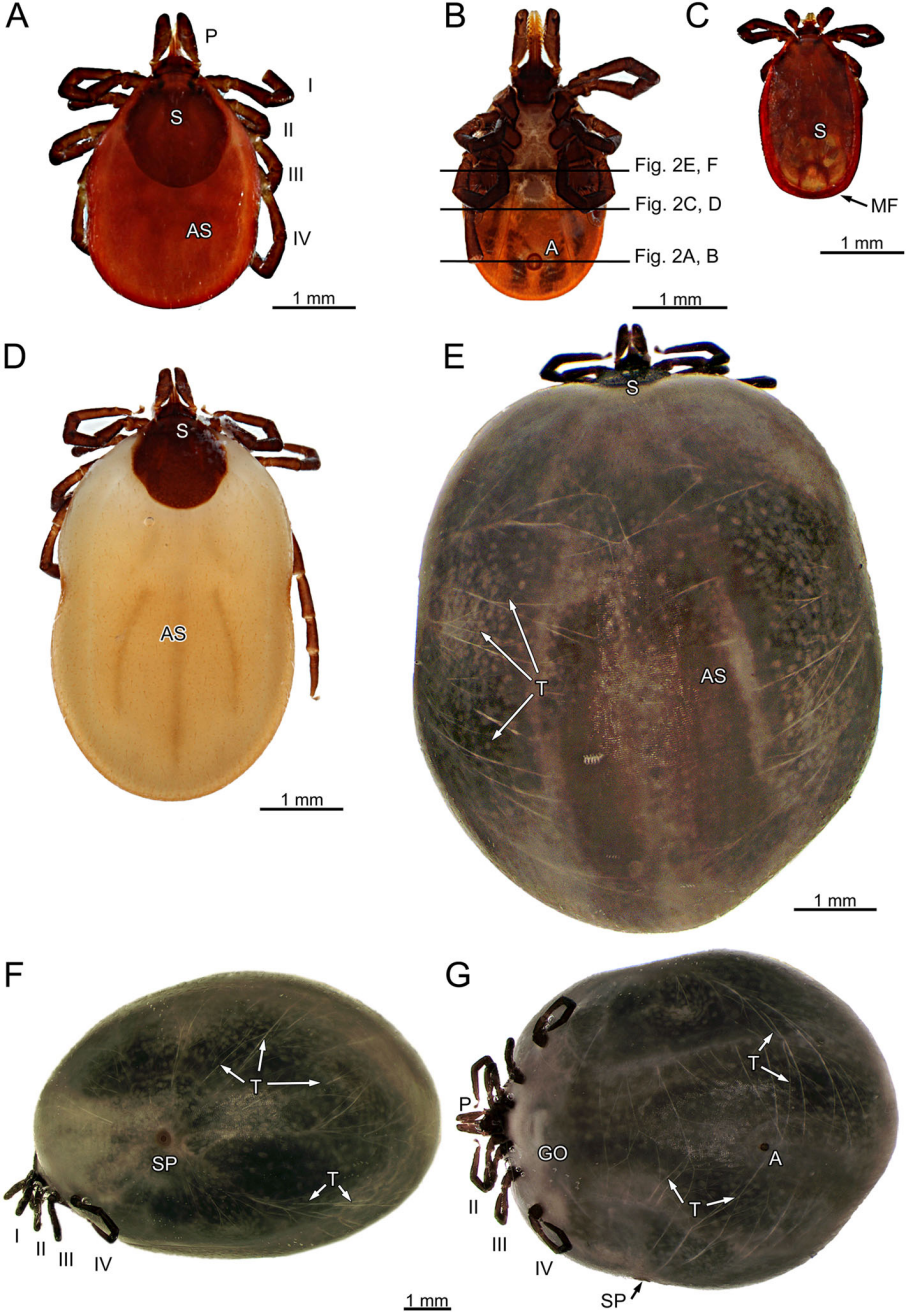
*Ixodes ricinus*. **a** Fasting female, dorsal view. **b** Fasting female, ventral view; black lines indicate positions of histological sections shown in Fig. 2a, c, and e and μCT-imaging Fig. 2b, d, and f, respectively. **c** Fasting male, dorsal view. **d** Semi-engorged female, dorsal view. **e** Fully engorged female, dorsal view. **f** Fully engorged female, lateral view. **g** Fully engorged female, ventral view. *Abbreviations*: A, anus; AS, alloscutum; GO, genital opening; MF, marginal fold; P, pedipalp; S, scutum; SP, spiracular plate; T, Tracheae; I – IV, legs

Adult females feed in two phases. The first phase lasts several days (4–5 days) and food intake is slow. The second phase is short (about 24 h) and food intake is rapid. During the slow feeding phase, tick volume increases about 10-fold as compared to fasting. During the rapid feeding phase, body volume increases another 10-fold or more, resulting in a total volume increase exceeding the fasting volume by the factor 100 [2–4]. This total volume increase of adult females is one of the most extreme short-term body volume changes among animals (Fig. 1).

Details of morphological responses to feeding have been described for cuticle and the digestive cells of the midgut epithelium of various species of hard bodied ticks (e.g., *Amblyomma cajennense, Boophilus microplus, B. decoloratus, Rhipicephalus appendiculatus, R. sanguineus, Hyalomma detritum, H. asiaticum, Ixodes ricinus*). However, many previous studies are descriptive and most studies focus on ultrastructural details (e.g., [5–11]). Functional aspects focus on specific details (e.g., [12–14]), but the overall tissue morphology is not considered. More recently, Remedio et al. [15] showed that the combined effects of increased mitotic activity and epidermal cell size (hypertrophy) contribute to an expansion of the epidermis of idiosoma of *R. sanguineus* during feeding. Generally, the current body of knowledge consists of detailed information scattered across species, but no organismic, functional, and mechanistic understanding of how ticks accomplish this enormous increase in body volume has been achieved. We use microscopic anatomical and histological methods to expand the existing body of knowledge by providing a new perspective on the morphology of female ticks, and new and explicit functional explanations for the reported results. By comparing fasting, semi-engorged, and fully engorged females, we study the effects of feeding associated volume increase on the morphology of (1) the epidermis and cuticle, (2) the epithelium of the midgut diverticula, and (3) the tracheae. Comparisons to the sexually dimorphic males support the functional interpretation, as males do not experience similar volume increases and, thus, any difference observed in the morphology of organs and tissues (cuticle, midgut diverticula, and tracheae) may be functionally relevant.

Given the more than 100-fold volume increase, a major challenge for the epidermis and the cuticle of ixodid ticks is providing sufficient area of the epidermal epithelium and the cuticle at the end of feeding to contain the expanded body. Expansion of folded cuticle, elastic properties, and secretion of new cuticle might serve as mechanisms supporting the necessary size changes. Indeed, several studies showed that the cuticle of the alloscutum of ixodid ticks expands by stretching cuticular folds and grows by cuticle secretion during both feeding phases [2, 5–8, 16]. Hypertrophy of epidermal cells has been reported [11, 14, 15] and supposedly accommodates the necessary expansion of the epidermal epithelium during the slow feeding phase. Cell division has not been observed in the epidermis of *Ixodes ricinus* [2], but in other ixodid species (*Hyalomma asiaticum*; 11, Balashov 1968 cit. in 14). Expandable properties of the cuticle of the alloscutum of ixodid ticks are located in the endocuticle, while the area of the exo- and epicuticle increases by unfolding [6, 12, 13]. The mechanism of endocuticle expansion has been described as “plasticization”, i.e., breaking of intermolecular non-covalent bonds, rather than elasticity [9, 17, 18]. However, Dillinger and Kesel [19] documented the occurrence of resilin in the deep valleys between the cuticular folds of the exocuticle, suggesting that mechanical unfolding together with an elastic component may account for the stretching of the outer cuticle layers.

Here, we ask how cells of the epidermal epithelium accommodate the size changes of the body surface. We hypothesize that an increase of the epidermal area is possible by: (i) cell proliferation (hyperplasia), (ii) increase of cell size (cellular hypertrophy), or (iii) by shape changes of the epidermal cells (e.g., from prismatic to squamous). These three possibilities are not mutually exclusive, and each alone, or any combination of the three, will result in an increase of the area of the epidermis. Because each of the suggested mechanisms would result in a different appearance of the cells of the epidermis in fasting, semi-engorged, and fully engorged ticks, histology is an appropriate and sufficient method for testing these hypotheses.

The blood engorged by castor bean ticks during feeding is partially digested, condensed and dehydrated, and a large volume of digesta is deposited in the diverticula of the midgut for absorption by the digestive cells. The wall of the midgut diverticula consists of the digestive epithelium and an external, incomplete layer of muscle cells. Previous studies on other ixodid species have reported cellular differentiation, hypertrophy, and hyperplasia of digestive cells. Based on studies on various mite species, it is generally accepted that resting digestive cells differentiate into functional cells that may have digestive, secretory and excretory functions [20–25]. Whether these different functions reflect different temporal stages of cellular differentiation of individual cells or entirely different cell types is unclear. How these changes accommodate volume changes of the midgut diverticula is also unresolved. We hypothesize that the necessary size changes of the midgut diverticula are accommodated by cellular hyperplasia, hypertrophy, or shape changes of the cells in the epithelium. Each of these suggested mechanisms results in different histological appearance of the cells; i.e., size increase of cells (e.g., by cellular growth and/or incorporation of nutrients), mitotic structures, and changes of the epithelial organization of the cells, respectively.

Volume changes of the midgut diverticula cause stretching and dislocation of other organ systems. In particular the tracheae, which are responsible for convective oxygen transport from the spiracular opening to sites of cellular respiration, must adjust to the expansion of the opisthosoma and bridge the increased length between the spiracular opening and the respiratory tissue. The continued functioning of the tracheae during all feeding phases is important because the hemolymphatic system is reduced and does not contain oxygen carrying proteins. The tracheae are the sole structures that provide convective oxygen transport to the respiratory tissue. We hypothesize that tracheae can adjust to the volume changes of the opisthosoma because (i) they are designed with substantial excess length resulting in a coiled topography of the tracheae in the fasting animals. During feeding, the tracheae would uncoil and the fully engorged tick would have straight tracheae. The distance between the taenidia of the tracheae should be constant in fasting and fed conditions, as the tracheae do not expand. Observing this will require the use of microscopic anatomical methods such as μCT-imaging and 3D-reconstruction to analyze the topography of tracheae in fasting and fully engorged ticks to test this hypothesis. (ii) Alternatively, the tracheae may be expandable and stretch along with the increase in body volume. In this case, we would find no excess material, but straight tracheae in fasting and in engorged animals. In that case, we predict that the distance between the taenidia of the tracheal wall would be narrow in fasting ticks, but wide in engorged ticks.

## Methods

*Ixodes ricinus* Linnaeus, 1758 were taken from the teaching collection of the Department of Biology, University of Munich. The collection contains 49 specimens preserved either in 4% paraformaldehyde or in 2.5% glutardialdehyde. For this study we used 36 specimens (five adult male, 14 fasting adult female, eight semi-engorged adult females, nine fully engorged adult females; according to label information only ticks attached to their hosts [domestic dogs] were collected). We also used one well preserved fasting specimen from a collection of KOH-macerated and mounted specimens for CLSM-imaging.

### Light microscopic histology

For light microscopy (LM), specimens were washed six times in phosphate buffered saline (0.1 mol l^− 1^) over a period of 24 h, with one step in a low vacuum to ensure that the tissue was free of air bubbles. Samples were then dehydrated through a graded series of ethanol (30–96%) with the final two steps in a low vacuum. The samples were embedded in hydroxyethyl methacrylate (Historesin; Leica Microsystems, Wetzlar, Germany). Histological sections were cut at 2 μm thickness using an AO Spencer No. 820 rotary microtome or a Microm HM 340 E electronic rotary microtome (Thermo Fisher Scientific Inc., Waltham, MA, USA). The sections were stained using Rüdeberg staining solution (0.1% methylene blue, 0.1% thionin and 0.1 mol l^− 1^ Na_2_HPO^4^ in distilled water; [26]). For histological analysis, we used either an (i) Olympus BX51TF microscope (Olympus, Hamburg, Germany) equipped with a microscope camera (UCMOS camera, ToupTek Photonics, Hangzhou, P. R. China) and image capturing software ToupView (ToupTek Photonics, Hangzhou, P. R. China), or (ii) an Olympus BX61VS scanning microscope equipped with an Olympus XC10 camera and VS-AWS FL 2.8 scanning and capturing software, or (iii) a Zeiss Axiophot equipped with an AxioCam ERc5s camera and Zen 2.3 blue edition (Zeiss 2011).

### Transmission electron microscopy

For transmission electron microscopy (TEM) we sampled small pieces from fasting and engorged specimens (*n* = 5 males fasting; *n* = 4 females fasting; *n* = 2 females fully engorged). Samples were washed four times in phosphate buffered saline (0.1 mol l^− 1^) over a period of 20 min, postfixed in 1% osmium tetroxide for two hours and washed again (4 times, 20 min each) in phosphate buffered saline to remove excess osmium tetroxide. Samples were dehydrated through graded series of acetone (30–100%) and then embedded in Epon (Carl Roth GmbH + Co. KG, Karlsruhe, Germany). Ultrathin sections were cut at 50 nm thickness using an RMC MTXL ultra-microtome (Boeckeler Instruments, Inc., Tucson, Arizona, USA). Sections were collected on copper triple slot grids and contrasted using uranyl acetate and lead citrate following standard protocols [27]. For TEM, we used a Morgagni 268 electron microscope (FEI Company, Hillsboro, OR, USA), and for image capture we used MegaView III CCD - iTEM-SIS software (Olympus, Soft Imaging System GmbH, Münster, Germany).

### Scanning electron microscopy

For scanning electron microscopy (SEM) we used glutardialdehyde (GDA 2.5% in 0.1 mol l^− 1^ phosphate buffered saline, pH 7.4) preserved specimens (*n* = 4 females; two fasting, two fully engorged). Specimens were washed three times in cacodylic acid sodium salt trihydrate buffer (0.1 mol l^− 1^; Carl Roth GmbH + Co. KG, Karlsruhe, Germany). For SEM of the tracheae in fasting and fully engorged ticks, we manually peeled off the cuticle so that underlying tracheae were exposed and partially broken. After postfixation with 1% osmium tetroxide for 30 min, the samples were washed again in cacodylic acid sodium salt trihydrate buffer three times. Dehydration was achieved with a graded series of acetone. The samples were then dried using the critical point drying method (Polaron Critical Point Dryer). The samples were mounted using Tempfix (Plano GmbH, Wetzlar, Germany) and later sputter coated for 60 s with gold using a BAL-TEC SCD 050 sputter coater (Leica Mikrosysteme Vertrieb GmbH, Wetzlar, Germany). Images were captured using a LEO 1430VP SEM (LEO Elektronenmikroskopie GmbH, Oberkochen, Germany) and SmartSEM software (version 5.07, Carl Zeiss AG, Oberkochen, Germany).

### Image processing

All LM, SEM and TEM images were processed using ImageJ (version 1.50d, NIH, USA). Image processing included contrast enhancing, flat-field and pseudo-flat-field correction. Assembly of images as well as addition of labels and scale bars were done using Adobe Illustrator CS2 (Adobe Systems Incorporated, San Jose, CA, USA).

Histological images were photographed either using the Olympus scanning microscope or manually at high resolution resulting in multiple microphotographs per image. For 2D-reconstruction of manually taken images we used the Image composition editor Version 2.0.3.0 (64 bit, Microsoft 2015).

### Macrophotography

We used a Canon EOS Rebel T3i equipped with a Canon MP-E 65 mm lens for macrophotography. Illumination was provided by two external flashlights with polarizing filters. We took multiple images in z-stacks at different focal levels. These images were later fused into one focal image using CombineZM (1.0.0). Color balance, saturation and sharpness were adjusted using Photoshop (Ver. 14.0).

### Confocal laser scanning microscopy

We used a Leica TCS SP confocal laser scanning microscope to produce autofluorescent images from cuticula preparations of a whole tick. To obtain optimal resolution the sample was scanned resulting in about 3500 individual images in x, y and z axes. Using Fiji [28] we produced multiple image stacks omitting images-levels of the ventral cuticle. Stacks were then reduced to one focal level. Using Photoshop (Ver. 14.0) these images were merged on the x and y axes [29].

### μCT-scanning

A fasting adult female and a fully engorged adult female fixed in paraformaldehyde were transferred to 70% ethanol, further dehydrated in a graded ethanol series and contrasted in 1% iodine solution (in 99.5% ethanol). Critical point drying was performed using a Leica EM CPD300 (Leica Mikrosysteme Vertrieb GmbH, Wetzlar, Germany). In preparation for μCT, the dried specimens were mounted with hot glue on an insect pin (size 1).

μCT-Scans were performed with an x-ray microscope (XRadia XCT-200, Carl Zeiss Microscopy GmbH, Jena, Germany) at the University of Greifswald, Zoological Institute and Museum. The XRadia XCT-200 is equipped with scintillator-objective lens units, which allow a flexible field of view. Scans were performed with 0.39× (fed tick) and 4× objectives (fasting tick) with X-ray source setting at 40 kV and 8 W for 7 s (fed tick) and 12 s (fasting tick) acquisition time. To record as much information as possible and due to the difference in size of the specimens, different objectives and settings were used for scanning.

Subsequently, 1600 projections per tomography were reconstructed with the XMReconstructor software (Carl Zeiss Microscopy GmbH, Jena, Germany), resulting in TIFF format image stacks. Both scans were performed using Binning 2 and subsequently reconstructed using Binning 1 (full resolution) to avoid information loss. The reconstruction resulted in system based calculated pixel size of 9.39 μm (0.39× objective), 1024 x 1024px, and system based calculated pixel size of 4.54 μm (4× objective), 1015 x 1015px. Consequently, the spatial resolution is optimally c. 9 μm for clear borders (e.g., cross section of a trachea) for the reconstructed TIFF-images based on the μCT of the fasting tick, or c. 15 μm for individual structures (e.g., cross section of a trachea), or 18 μm and 28 μm for the reconstructed TIFF-images based on the μCT of the fed tick.

### 3D-reconstruction

The body surface and the tracheal system were reconstructed using surface rendering in Amira 6.0.0 (Mercury Computer Systems Inc., Chelmsford, MA, USA). The body surface and the tracheae of each μCT-image were labelled using a graphic pen; all tracheae were marked in red. Selected μCT-images and respective labeling are provided in supplementary online material (Additional file 1: Figure S1) to document how segmented images translate into raw data for the 3D-reconstruction.

## Results

Volume changes of ticks are evident. Figure 1 shows a fasting female (Fig. 1a, b), a fasting male (Fig. 1c), a semi-engorged female (Fig. 1d), and a fully engorged female (Fig. 1e–g). Fasting females (4 mm total body length) are larger than fasting males (2.5 mm total body length). The idiosoma of females is dorsally covered by the sclerotized scutum (S) and the soft alloscutum (AS, Fig. 1a); on the ventral side it is covered by soft alloscutum and the sclerotized coxae of the legs (Fig. 1b). Only the parts of the body covered by alloscutum extend during feeding whereas the scutum does not change in size. In males, the dorsal scutum extends to the posterior margin of the idiosoma (Fig. 1c). The ventral side of the male body is covered by several sclerites (pregenital, median, anal, adanal and epimeral plates), the pregenital and the median plate reaching between the coxae of the legs. A thin marginal fold (MF) of flexible cuticle connects scutum and ventral plates (Fig. 1c). Figure 1e–g show a fully engorged female in dorsal, lateral and ventral view documenting the expansion of the regions covered by soft alloscutum. In lateral view, the spiracular plate (SP) is found in the anterior third of the opisthosoma. Superficial tracheae (T) are clearly visibly expanding radially from the spiracular plate. The genital opening (GO) and the anus (A) are seen in ventral view in the midline of the opisthosoma.

### Microscopic anatomy

**Fig. 2:**
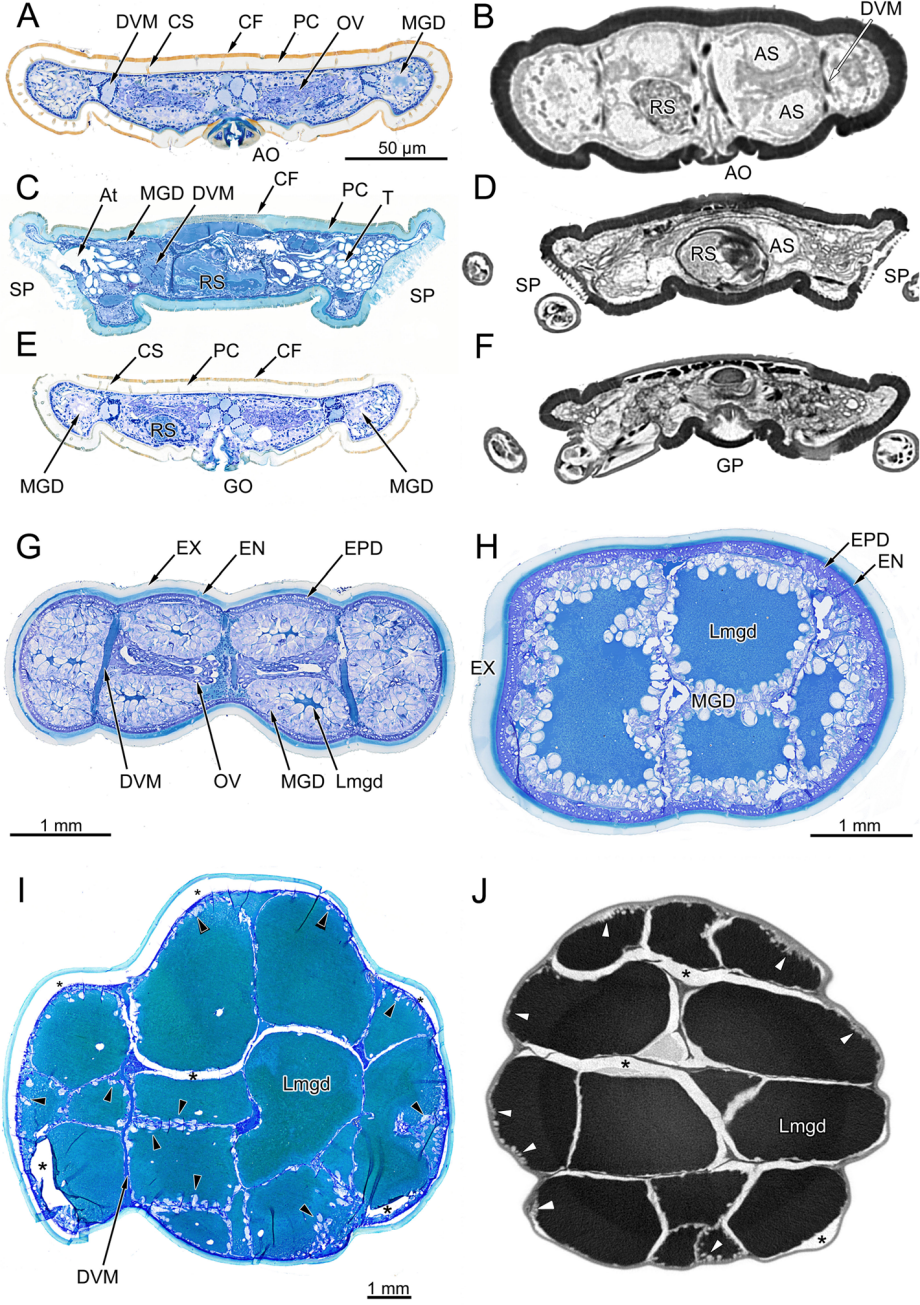
*Ixodes ricinus*, microscopic anatomy of fasting, semi-engorged, and fully engorged females. The plate contrasts micrographs of histological cross-sections with μCT-images. **a** Histological micrograph of a cross-section at the level of the anal opening. **b** Grey-level inverted μCT-image at same position as in (**a**). **c** Histological micrograph of a cross-section at the level of the spiracular plates. **d** Grey-level inverted μCT-image at same position as in **c**. **e** Histological micrograph of a cross-section at the level of the genital opening. **f** Grey-level inverted μCT-image at same position as in **e**. Figures **a**, **c**, **e** are from same individual as in Fig. 1b. **g** Semi-engorged female (previous feeding stage as compared to H), histological micrograph of a cross-section in the posterior region of the opisthosoma, same individual as Fig. 1d. **h** Semi-engorged tick, histological micrograph of a cross-section in the posterior region of the opisthosoma. **i** Fully engorged tick, histological micrograph of a cross-section in the posterior region of the opisthosoma. **j** Grey-level inverted μCT-image at same position as in “**i**”. *Abbreviations:* AO, anal opening; AS, anal sac; At, atrium; CF, cuticular folds; DVM, dorso-ventral musculature; EN, endocuticula; EPD, epidermis; EX, exocuticula; GO, genital opening; GP, genital plate; Lmgd, lumen of a midgut diverticulum; MGD, midgut diverticulum; OV, ovary; PC, procuticle; RS, receptaculum seminis; SP; spiracular plate; T, tracheae. Arrowheads in “**i**” and “**j**” point to the digestive cells of the midgut epithelium. Figures **a**–**f** same scale

**Fig. 3:**
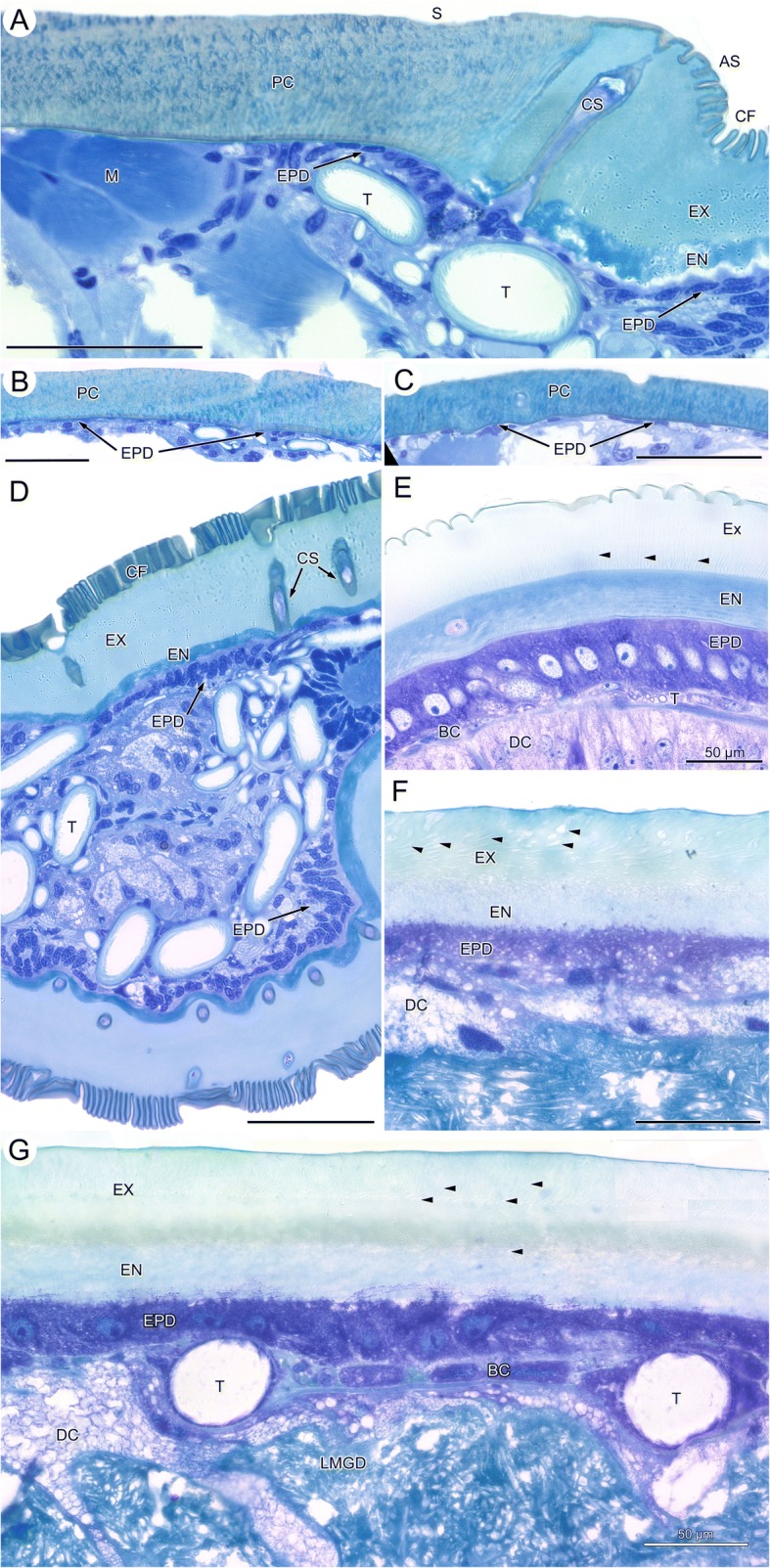
*Ixodes ricinus*, histological micrographs of the cuticle in fasting, semi-engorged and fully engorged condition. **a** Adult female in fasting condition, transition between scutum and alloscutum. **b** Adult female, fasting, cuticle of the scutum. **c** Adult male, cross section through the opisthosoma with cuticle of the dorsal scutum covering the body. **d** Fasting female, section through the lateral region of the opisthosoma showing dorsal and ventral alloscutum, and cross-sections through midgut diverticula and tracheae. **e** Semi-engorged female, section through the alloscutum. **f** Fully engorged female, high power light micrograph of a section through the alloscutum. **g** Fully-engorged female, section through the cuticle of the alloscutum, epidermis and midgut epithelium. Note two large tracheae squeezed between midgut epithelium and epidermis. *Abbreviations*: AS, alloscutum; BC, basal cells of midgut diverticula; CF, cuticular folds; CS, cuticula sensillum; DC, digestive cell; EN, endocuticula; EPD, epidermis; EX, exocuticula; LMDG, lumen of midgut diverticulum; M, muscle; PC, procuticle; S, scutum; T, trachea. Arrowheads indicate pore canals. In Figures **a**-**g**, scale bar = 50 μm

Microscopic anatomical changes associated with food intake are shown in cross-sections through the opisthosoma of fasting ticks, semi-engorged, and fully-engorged ticks. In low power overview micrographs of fasting ticks, the cuticle of the alloscutum has an outer margin of cuticular folds (CF; Figs. 2, 3a, d) a thick procuticle (Figs. 2, 3a; PC), and relatively large canals for cuticular sensilla (Figs. 2, 3a, d; CS). The cells of the underlying epithelium of the epiderm are small (Fig. 3a-c; EPD;). Due to the lower resolution, these details are not seen in μCT-images in which the cuticle appears homogeneous; cuticular folds are not resolved and the canals of cuticular sensilla are seen only as faint differences in grey level (Fig. 2b, d, f). The layer of epidermal cells cannot be seen in μCT-images.

In low-power histological micrographs of semi-engorged ticks, the cuticular folds are smoothed, and the cuticle now shows a differentiation into an internal, intensively staining endocuticle and an external exocuticle. The cells of the epidermal epithelium are also enlarged and are recognizable as a single layered epithelium of large prismatic cells, even at low magnification (Figs. 2g–h, 3e). In cross-sectional overview images of fully engorged ticks, we could no longer recognize these details of the cuticle and epidermis. This is mainly due to the low-power magnification needed to obtain an overview image of fully engorged ticks (Fig. 2i–j).

The midgut diverticula (MGD) of fasting ticks are rudimentary, blind ending tubes. They have barely a lumen, only the lateral and terminal parts of the midgut diverticula may have a small lumen. Midgut diverticula extend through the opisthosoma and are found in posterior sections within the proximity of the anal opening (Fig. 2a, b), more anteriorly at the level of the spiracular plate (Fig. 2c, d), or at the level of the genital opening (Fig. 2e, f). In histological cross-sections the midgut diverticula are diagnosed by the epithelium of degenerative and resting digestive cells (Fig. 5a). Such cytological detail is missing in μCT-images; thus, midgut diverticula can be diagnosed on μCT-images only by their topographic position. However, because other organ system (e.g., anal sac, gonads, and salivary glands) show a similar appearance in μCT-images and are in the topographic proximity, the diagnostic features of µCT-imaging are considered insufficient without the help of parallel histological slides.

The midgut diverticula of semi-engorged ticks (Fig. 2g, h) have obtained a considerable size. In particular the cells of the midgut epithelium are large and club shaped, extending into the lumen of the midgut diverticula (Fig. 2g, h). The size of digestive cells of the midgut epithelium may exceed 100 μm in basal to apical length as well as diameter. As seen in Fig. 2, the lumina of numerous midgut diverticula of fully engorged ticks fill virtually the entire cross section of the opisthosoma (Fig. 2i, j), forcing all other organ systems laterally or to the outer margin. The cells of the digestive epithelium have enlarged enormously now reaching more than 50 μm diameter and thus can be recognized even in low power micrographs and in μCT-images (Fig. 2i, j; black/white arrow-heads). In both imaging methods, tissue preparation creates shrinking artefacts (Fig. 2i, j asterisks) preventing a flawless and undisturbed evaluation of the microscopic anatomy. The critical point drying for μCT-imaging results in relatively large, low-contrast, air-filled spaces around the midgut diverticula.

Tracheae, specifically tracheoles are too small to be seen in low-power micrographs. Only the spiracular plates, the atrium and large stem tracheae can be seen in the histological cross-sections (Fig. 2c) and μCT-images (Fig. 2d) documenting the intense convolute of tracheae immediately below the atrium.

### Histology of the epidermis and the cuticle

In light microscopy, the epidermis (EPD) of the female scutum forms a squamous epithelium of thin and elongate cells overlain by a procuticle (Fig. 3a, b; PC). Here, the procuticle is about 60–80 μm thick, and it is not possible to differentiate between endocuticle and exocuticle. The procuticle stains heterogeneously, indicating differences in density. The epicuticle is too thin to be visualized using light microscopy. In light microscopy, pore canals cannot be seen, but many sections of the scutum and the alloscutum contain large pores of cuticular sensilla. In males, the epidermis and the cuticle (Fig. 3c) of the dorsal and the ventral sclerites have the same histological structure as described for the scutum of females (Additional file 2: Figure S2).

**Fig. 4:**
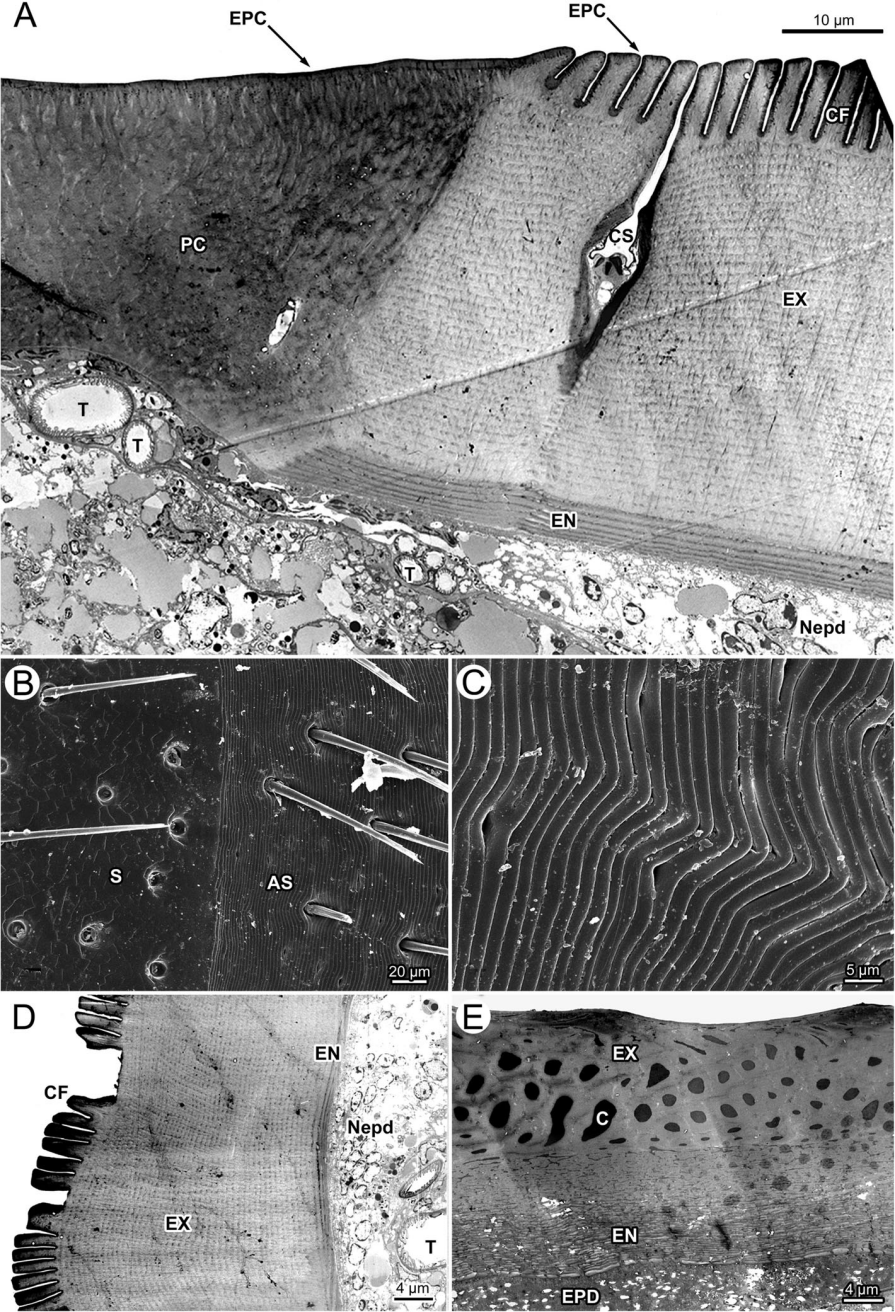
*Ixodes ricinus,* electron microscopic micrographs of the cuticle. **a** TEM of the cuticle of a fasting female. The image shows the transition between scutum (left) and alloscutum (right). **b** SEM of the transition from scutum (left) to alloscutum (right) of a fasting female. **c** SEM of the cuticle of the alloscutum of a fasting female; close-up of the cuticular folds. **d** TEM of the alloscutum of a fasting female. **e** TEM of the cuticle of a fully engorged female. *Abbreviations:* AS, alloscutum; C, pore canal; CF, cuticular folds; CS, cuticular sensilla; EN, endocuticle; EPC, epicuticle; EPD, epidermis; EX, exocuticle; Nepd, nuclei of epidermal cells; PC, procuticle; S, scutum; T, trachea

The following description is for the scutum and the underlying epidermis cells of unfed and fed, male and female *Ixodes ricinus* ticks. Transmission electron microscopy of the scutum of fasting females confirms a procuticle without differentiation into endo- and exocuticle (Fig. 4a). A thin, electron-dense epicuticle (EPC) covers the cuticle superficially. The procuticle is organized in packages of electron-dense and less electron-dense areas resulting in a heterogeneous appearance. Numerous sections through thin pore canals are found in TEM micrographs of the scutum (Fig. 4a). Beneath the scutum, the epidermal cells are arranged as a squamous epithelium with thin, flattened epidermal cells. Their histological appearance does not change during the feeding cycle.

The transition between scutum and alloscutum is abrupt (Figs. 1, 3a, 4a, b). The epithelial cells of the alloscutal epidermis and the structure of the cuticle differ from those of the scutum. The surface of the alloscutum is characterize by apical folds that are about 10 μm high and 2 μm thick (Figs. 3a, d, 4a). In SEM, these folds are more or less straight, but occasionally form a shallow wave pattern (Fig. 4b, c). In light microscopy the cuticle of the alloscutum is differentiated into an inner, intensively staining endocuticle and an outer, pale exocuticle (Fig. 3a, d). In TEM, the cuticle of the alloscutum of fasting female ticks shows a thin and layered endocuticle (EN), a thick, layered exocuticle (EX), and a thin, apical layer of electron dense epicuticle (Fig. 4a, d; EPC). The endocuticle and the exocuticle are layered in numerous strata, with some faint lines oriented perpendicular to the strata, probably representing pore canals (Fig. 4a, d).

The epidermal cells of the alloscutum have a small cytoplasmic compartment and a relatively large elongate oval nucleus. The long axis of the nuclei of the epidermal cells is oriented perpendicular to the cuticle. Nuclei are enlarged compared to those of epidermal cells beneath the scutum (Fig. 3a, b). In many positions the epidermal cells are arranged as a prismatic epithelium or frequently they are arranged in several layers (pseudostratified epithelium; Fig. 3d), suggesting that they are overlaying each other. Transmission electron microscopy confirms the findings of a pseudostratified epidermis (Fig. 4a, d; Nepd) of the alloscutal cuticle.

Male ticks have no alloscutal cuticle, but marginal folds that accommodate minor change of body volume (Fig. 1c; Additional file 2: Figure S2). The cuticle of these marginal folds has the same cytological appearance as described for the scutum in females, but in the bent regions of the marginal folds the cuticle is thinner and less stained, suggesting a lower degree of sclerotization. No apical cuticular folds are found but a relatively thick epicuticle as compared to the scutum of females. The epidermis underlying the cuticle of the marginal folds is a thin squamous epithelium (Fig. 3c).

In semi-engorged females, we observe prominent cytological differences of cuticle and epidermis compared to fasting ticks. The cytoplasmic compartment and the nuclei of the epidermal cells have increased manifold (Fig. 3e; Additional file 3: Figure S3). The cells of the epiderm are now arranged as a single layered epithelium of large prismatic cells with a central, round to ovoid nucleus. The nucleus contains mainly heterochromatin but has one, sometimes two, small round nucleoli.

Also, the apical surface of the cuticle has stretched and the cuticular folds now form a shallow and wavy surface (Fig. 3e, Additional file 3: Figure S3). The cuticle is thicker, in particular the endocuticle, than in fasting ticks. It shows a heterogeneous loose structure. Within the exocuticle the pore canals are now recognizable in light microscopy. It should be noted that we did not find a single mitotic structure in the epidermal cells of semi-engorged ticks, suggesting that epithelial structure, as well as the size and the shape of cells change by cellular differentiation and cellular hypertrophy rather than hyperplasia.

In fully engorged female ticks, the epidermis forms a cuboidal to squamous epithelium, with relatively large cells containing large round central nuclei, but the appearance of the epithelial cells has changed to flattened shape (Fig. 3f, g). The apical border of the cells is undefined and cytoplasmic extensions appear to reach into the cuticle. The pore canals are enlarged and recognizable in the endocuticle as well as exocuticle. The surface of the alloscutal cuticle is completely flat. Transmission electron microscopy documents proportional differences in the thickness of endocuticle and exocuticle. Both show about the same thickness, suggesting more stretching of the exocuticle as compared to the endocuticle (Fig. 4e). The lumen of the pore canals (C) in the exocuticle is enlarged and filled with an electron dense material (Fig. 4e). The endocuticle maintains a layered structure (Fig. 4e).

### Midgut diverticula

**Fig. 5:**
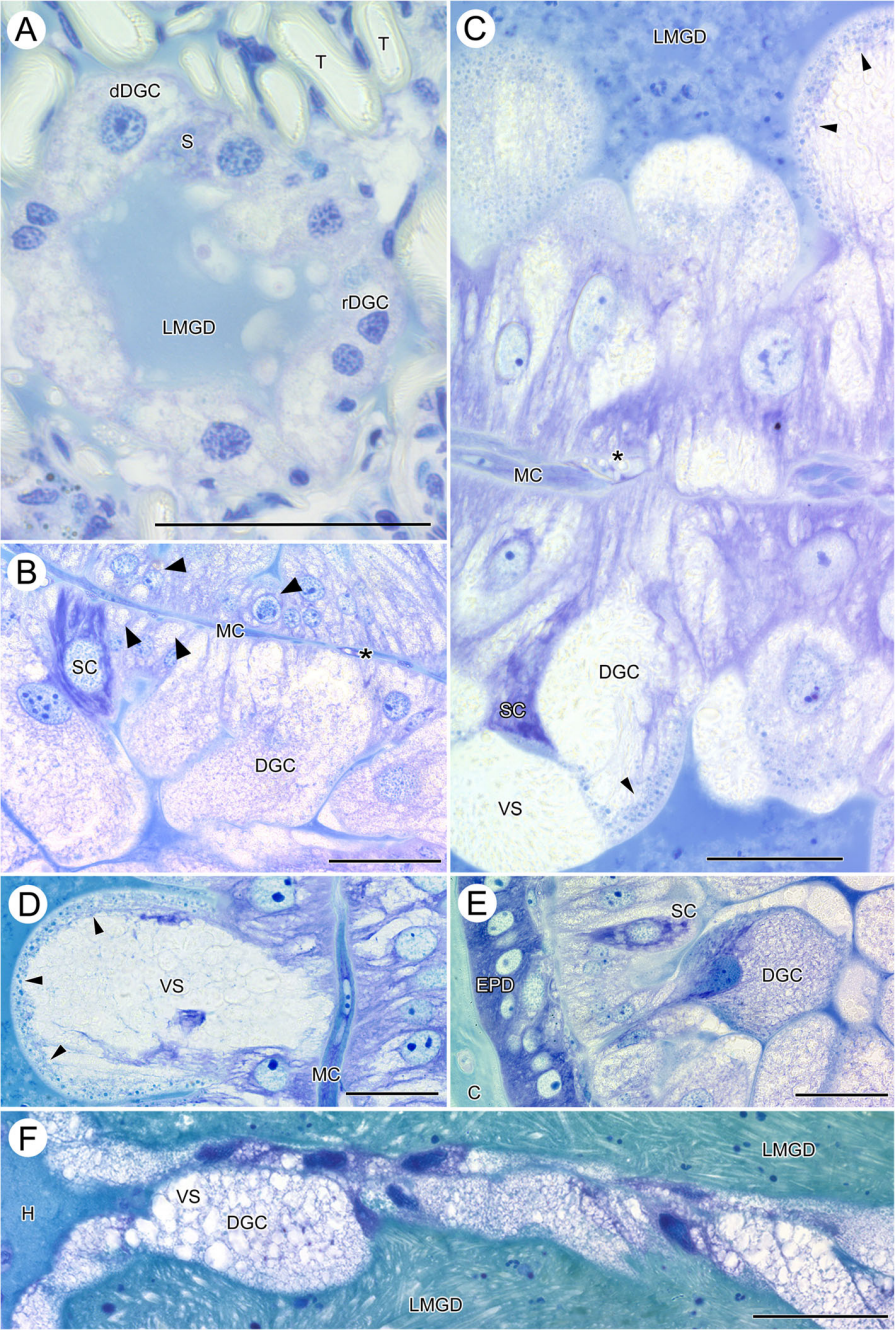
*Ixodes ricinus,* LM micrographs of the midgut diverticula in fasting, semi-engorged, and fully engorged females. **a** cross-section through a midgut diverticulum of a fasting tick. **b** Epithelium of two neighboring midgut diverticula of an early feeding stage tick. **c** Epithelium of two neighboring midgut diverticula of a semi-engorged tick. **d** High-power micrograph of an individual digestive cell in the epithelium of a midgut diverticulum of a semi-engorged tick. **e** High-power micrograph of the epithelium of a midgut diverticulum directly under the epidermis (**f**) Epithelium of two adjoining midgut diverticula of a fully engorged tick. *Abbreviations*: C, cuticle; dDGC, degenerative digestive cell; DGC, digestive cell; EPD, epidermis; H, hemolymphatic space; LMGD, lumen of the midgut diverticula; MC, muscle cell; rDGC, resting digestive cell; S, stem cell; SC, secretory cells; T, trachea; VS, vesicles; arrowheads point to the marginal zone of small, intensively staining vesicles. All scale bars 50 μm

In fasting ticks, the midgut diverticula are small tube-like extensions from the midgut that have no visible lumen. Only the distal parts of the lateral diverticula may show a small open lumen in fasting condition (Fig. 5a; LMGD). The epithelium of the midgut diverticula of fasting ticks is a single layered epithelium of resting digestive cells (rDGC), degenerative digestive cells (dDGC), and stem cells (S). In light microscopy, degenerative digestive cells are comparatively large cells with a large round nucleus intensively speckled with spots of euchromatin and weakly staining cytoplasm that is filled with numerous vesicles. Resting digestive cells are distinctly smaller cells with irregular shaped nucleus. Their cytoplasmic compartment contains fewer and smaller vesicles than in degenerative digestive cells. Stem cells are small, intensively staining cells located between both other cell types (Fig. 5a). A thin and incomplete layer of basal cells surrounds the midgut epithelium at its basis. The cells of this layer are extremely thin and can be detected mainly by their elongate nuclei. No diagnostic features of these cells are differentiated in fasting ticks.

In semi-engorged female ticks, the epithelial cells of the midgut diverticula have increased in size and shape. The digestive cells are now club-shaped and many-fold increased in size (> 50 μm) as compared to digestive cells in fasting ticks (Fig. 5b–e). The enlarged, balloon-shaped apical part of the cells reaches into the lumen of the diverticula, while the thin basal pole of the cells roots on the basal membrane. The nucleus of the club-shaped cells is also enlarged, contains several nucleoli and is speckled with euchromatin. The apical part of the club-shaped cells is filled with numerous vesicles of different size and staining properties. Smaller digestive cells reside beside these enlarged cells, but, despite the size, they show the same cytological characters (Fig. 5c, d; black arrowheads). A second type of cells characterized by intensely purple staining cytoplasm with lighter spots and a large pale nucleus with only one nucleolus are found between the digestive cells; we consider those secretory cells (Fig. 5b, c, e; SC). The lumen of the midgut diverticula is still relatively small but stains intensively. The filling material is not cellular. The basal layer of cells surrounding the midgut diverticula is differentiated as compared to fasting ticks. These cells are enlarged, nuclei are elongate oval and their thin layer of cytoplasm has the typical appearance of muscle cells. Thus, the cells may have differentiated into a circular layer of muscle cells (Fig. 5b–d; MC). External to these basal cells, numerous small tracheae are found attached to either these basal cells or directly to the basal membrane of the epithelial cells (Fig. 5b, c; asterisks). The midgut diverticula are surrounded by small residues of the hemolymphatic space. In larger but not fully engorged ticks (thus probably somewhat later feeding stages), the club-shaped cells are further increased in size and reach far into the lumen of the midgut diverticula. These cells show a differentiation into a marginal zone containing small but intensively staining vesicles, and a more central part with larger but poorly staining vesicles (Fig. 5d; black arrowheads). In semi-engorged ticks, the midgut epithelium is a multilayered pseudostratified epithelium with all cells attaching to the basal extracellular matrix, but with the cell bodies arranged in several layers on top of each other. Some of these cells may be seen only with their apical or basal parts (Fig. 5c, e). This is clearly a combined effect of very large cell size and thin sectioning.

The lumen of the midgut diverticula now shows corpuscular structures of the contents, supposedly partially/fully digested erythrocytes from the prey (Fig. 5c).

In fully engorged ticks, the digestive cells are stretched (Fig. 5f), and in the extreme, may take the shape of a thin squamous epithelium. Figure 5f shows the epithelia of two neighboring midgut diverticula. In the upper, the cells are stretched to a thin squamous epithelium, while in the lower the cells are stretched but still bulge a bit in the lumen of the midgut. However, in fully engorged ticks we always find the epithelium of the midgut diverticula as a single layered epithelium in which the cells show different degrees of stretching (see also Additional file 4: Figure S4). The cytoplasmic compartment of the digestive cells is filled with numerous vesicles, the nuclei stain intensively throughout, no heterochromatin and no nucleoli can be recognized. They have lost the club shaped form. The surrounding layer of basal muscle cells can be seen only in few places. The lumen of the midgut diverticula is filled with “crystalline” digesta, no corpuscular structure was observed (Fig. 5f, Additional file 4: Figure S4).

### Respiratory system

**Fig. 6:**
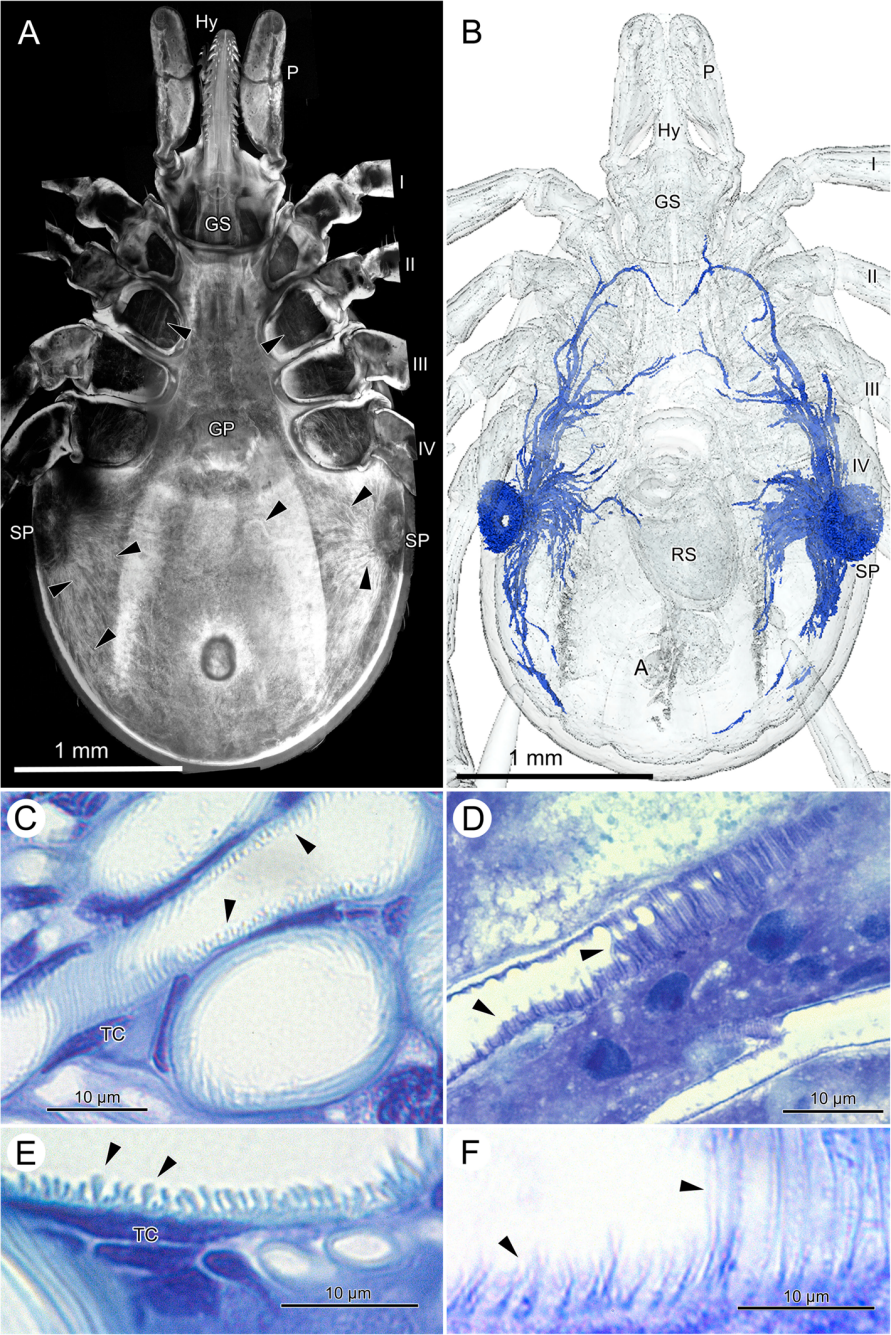
*Ixodes ricinus,* cross morphology and histology of the tracheal system. **a** Autofluorescent CLSM image of the tracheal system, macerated specimen; ventral view; arrowheads indicate tracheae. **b** 3D-reconstruction of the major tracheal trunks based on μCT-image stacks; ventral view. Tracheae are modeled in blue. The spatial resolution of the μCT-images is about 15 μm to recognize individual tracheae, therefore only tracheae larger than 15 μm are shown. **c** LM micrograph with longitudinal and cross-sectional sections of tracheae from a fasting animal; arrow-heads indicate taenidia. **d** LM micrograph with longitudinal sections of tracheae from a fully engorged animal. Note that the taenidia are almost completely flattened as compared to (**c**); the un-stretchable core of exocuticle in the taenidia can be recognized as a straight lightly stained line; arrowheads indicate taenidia. **e** High-power LM micrograph of the tracheal wall, showing the taenidia in an unfed tick. **f** High-power LM micrograph of the tracheal wall, showing the taenidia in a fully engorged tick. *Abbreviations:* A, anus; GP, genital plate; GS, gnathosoma; Hy, hypostome; P, pedipalp; RS, receptaculum seminis; SP, spiracular plate; TC, tracheal cell; I–IV, legs

Ticks have an extensive tracheal system that opens through two lateral spiracular plates (Figs. 2c, d, 6a, b). From a tracheal atrium under the spiracular opening, a high number of tracheae emerges and reaches through the entire body (Fig. 6a, b). In CLSM-images, the autofluorescence of the cuticular intima of the tracheae provides a remarkably detailed image even from macerated specimens (Fig. 6a). However, in fasting female ticks, the tracheae are so dense that it is basically impossible describing the topographic anatomy of the tracheae. At CLSM-resolution (Fig. 6a) and in the μCT-based 3D-reconstructions the course of the tracheae from the atrium under the spiracular opening to the periphery is straight.

Micro-CT imaging and 3D-reconstruction of the tracheae are straightforward methods to analyze topographic anatomy, but, in case of the respiratory system of ticks, are limited by the spatial resolution (here we reached a minimum voxel size of 5.5 μm resulting in spatial resolution of ca. 15-20 μm). Therefore, Fig. 6b shows only the relatively large convective tracheae originating with a diameter of about 20 μm extending from the tracheal atrium to the periphery. Note that the μCT-based reconstruction fails to detect tracheae reaching to the midline of the body (Fig. 6b), trachea that can be seen in the CLSM image (Fig. 6a). However, CLSM image and 3D-reconstruction document that all tracheae of fasting female ticks are straight or only slightly curved, no tracheal coils are visible.

**Fig. 7:**
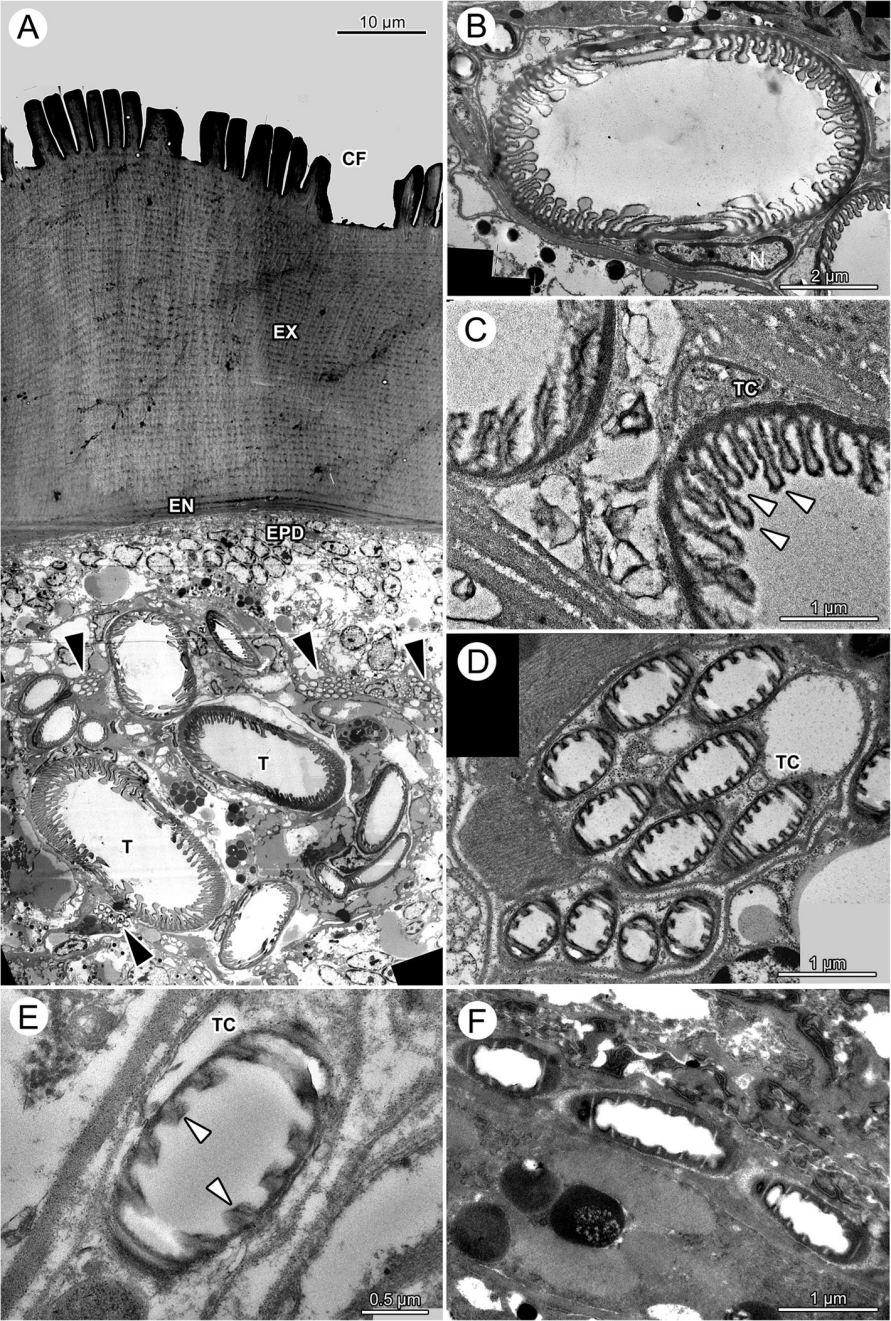
*Ixodes ricinus,* TEM of tracheae and tracheoles. **a** Low-power, overview micrograph of larger and smaller tracheae of a fasting tick positioned directly under the cuticle. Note that the large tracheae reside in the hemolymphatic space while bundles of tracheoles are densely packed in tracheal cells and connected to other tissues. Arrowheads point to individual tracheae. **b** Cross section through a large trachea of a fasting tick. The inner cuticular lining of the tracheae forms taenidia of variable height and shape. In particular, the cross section of the larger taenidia is club shaped. **c** Detail of the taenidia of two neighboring tracheae of a fasting tick. The electron-dense epicuticle forms irregular surface extensions. **d** Cross-section through two bundles of tracheoles of a fasting tick, each bundle residing in its own tracheal cell. Tracheoles show regular taenidia. **e** High power magnification of a single tracheole in a fasting tick. **f** High power magnification of a single tracheole in a fully engorged tick. Note that the taenidia have disappeared suggesting stretching of the tracheoles. The electron-light core of the taenidia is still recognizable as light stripes, suggesting that it is mainly the intertaenidial region that stretches when ticks increase in volume. Abbreviations: CF, cuticular folds; EN, endocuticle; EPD epidermis; EX, exocuticle; N, nucleus of tracheal cell; T, trachea; TC, tracheal cell; black arrowhead, bundles of tracheoles; white arrowheads, taenidia

On their course to the periphery, larger tracheae are always found in the (largely reduced) hemolymphatic space. Small tracheae and tracheoles attach to the organs and tissue, and have a tight cellular contact. In fasting ticks, we find bundles of small tracheoles with diameter below 1 μm (Fig. 7a, d).

We did not create a 3D-reconstruction of the tracheal system of a fully engorged tick, as we could obtain a voxel size of only 9.39 μm with a resulting spatial resolution of c. 28 μm. This resolution was not sufficient even for the major tracheae. However, freshly preserved ticks allow detecting the superficial tracheae positioned immediately below the cuticle and epidermis, using standard dissecting scopes (Fig. 1e–g). These surface tracheae originate from the spiracle and radially stretch over the enlarged body. The visible ends of these subcuticular tracheae reach to the dorsal and ventral midline of the opisthosoma. Given the size changes of the opisthosoma, the tracheae must have extended considerably (compare Fig. 1e–f with Fig. 6b). We could not observe the topographic changes of the deeper tracheae supplying the internal organs. However, given the size changes and given the fact that tracheoles are firmly fixed to the respiring tissues and organs, we assume a similar extension of the more interior tracheae.

**Fig. 8:**
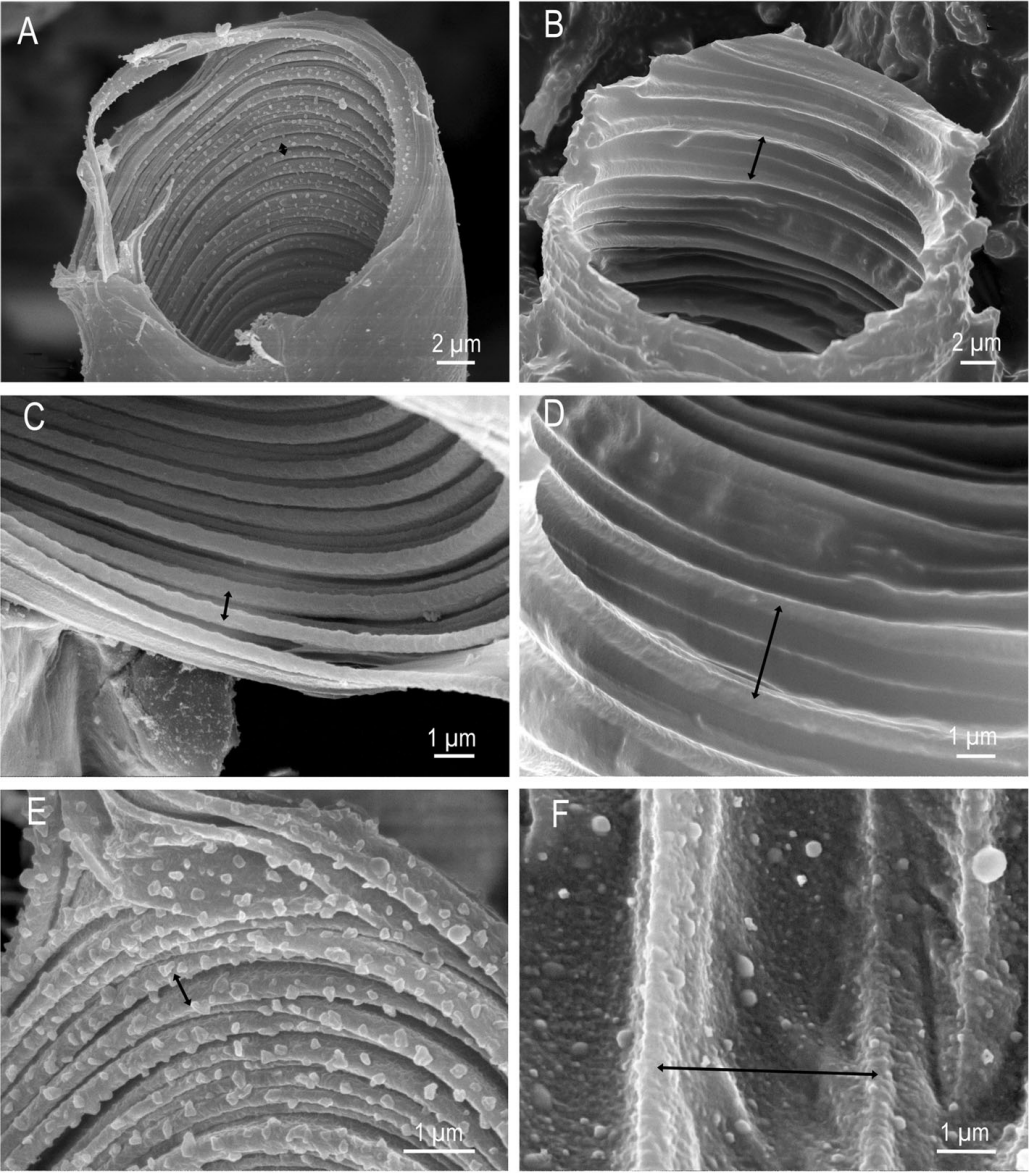
*Ixodes ricinus,* scanning electron micrographs of convective tracheae from fasting (left image column, figures **a**, **c**, **e**) and fully engorged (right image column, figures **b**, **d**, **f**) females. **a** Low power micrograph of a trachea of a fasting tick. The distance between the taenidiae is minimal. **b** Low power micrograph of a trachea of a fully engorged tick. Same magnification as in (**a**). The distance between the taenidiae has greatly extended (black arrow). **c** Trachea of a fasting tick at intermediate magnification. **d** Trachea of a fully engorged tick at intermediate magnification. The distance between taenidia has more than tripled, indicating the expansion of the tracheae associated with the volume increase of the body. **e** High power magnification of a trachea of a fasting tick. In this position the internal surface of the tracheae is densely covered with microstructures. **f** High power magnification of a trachea of a fully engorged tick. Same magnification as in (**e**). Double headed arrows indicate distance between two neighboring taenidia in fasting (left) and digesting (right) ticks

The fine structure of tracheae of fasting ticks shows a tracheal cell in which a single trachea is embedded (Fig. 6c, e; 7a–c). The trachea itself consists of three layers, an outer, electron-dense endocuticle, a translucent exocuticle forming the core of the taenidia, and an electron-dense epicuticle that covers the inner surface of the trachea (Fig. 7). The exocuticle is found exclusively in the core of the taenidia. The epicuticle forms micropapillae reaching into the lumen of the trachea (Figs. 7c; 8e, f). The taenidia are variable in size and shape (Figs. 6e, 7a–c). In cross-sections, large club-shaped taenidia alternate with smaller, lamellate taenidia (Figs. 7a–c; 8c, d). Small tracheae (i.e., tracheoles) with a diameter smaller than 1 μm occur in bundles of several tracheoles within one tracheal cell (arrowhead in Fig. 7a, d). The principle structure of the tracheal wall is the same as described above, with an electron-dense endocuticle, electron-translucent exocuticle (in the core of the taenidia), and an electron-dense epicuticle. The exocuticle occurs only in the core of the taenidia. The shape of the taenidia is more regular than in the larger tracheae. It should be noted that the taenidial structure of the inner lining of the tracheae can be readily seen in cross-sections and longitudinal sections of fasting ticks in light and transmission electron microscopy (Figs. 3a, d, 6c, e, 7a–e).

The structure of the cuticular lining of the tracheae changes with feeding. In light microscopy, longitudinal sections through tracheae of fully engorged ticks show flattened taenidia and a larger distance between them as compared to fasting ticks (Fig. 6d, f). While we counted about 12 taenidia per 10 μm in fasting ticks, we observed only 5–6 taenidia in tracheae of the same size in fully engorged ticks. In cross-sections of tracheae of fully engorged ticks, the taenidia are reduced to shallow waves of the inner surface or have completely disappeared (Fig. 3g). Tracheoles as smallest respiratory units cannot be detected in light microscopy. Using TEM, tracheoles of fully engorged ticks show distinctly flattened taenidia (Fig. 7f). The intertaenidial endocuticle (procuticle) has stretched, while the exocuticle in the core of the taenidia remains more or less unchanged and is recognizable as a narrow electron-translucent stripe.

Scanning electron microscopy of tracheae from fasting and fully engorged ticks confirm these results. It is evident from the series of scanning electron micrographs presenting different tracheae in fasting (Fig. 8a, c, e) and in fully engorged condition (Fig. 8b, d, f) that the distance between the taenidia increases three to five times. The cumulative increase in intertaenidial distance contributes to the overall extension of the entire trachea.

## Discussion

The aim of this study was to analyze morphological changes of different organ systems of the castor bean tick in response to food intake and volume increase during feeding. The study focuses on configuration changes of the cells of the epidermal epithelium, the midgut diverticula, and the tracheae, because these three organ systems immediately follow the volume changes of the opisthosoma during feeding.

The focus of this study was on the shape of the epidermal cells of the alloscutum and the configuration of the epithelium. Both undergo considerable changes during feeding. In fasting ticks, the epidermal cells are small and have only little cytoplasm. The general appearance of the epidermal cells is that of an undifferentiated almost embryonic tissue. They are packed so densely, that their nuclei are arranged in several layers under the cuticle resulting in a pseudostratified epithelium. Comparisons of the epidermal cells of the alloscutum with those of the scutum show that under the scutum the epidermis forms a squamous epithelium and that the cells do not overlay each other. Dorsal and ventral sclerites in male ticks also have a single-layered, squamous epithelium of the cuticle. This comparison supports the interpretation that the pseudostratified arrangement of the epidermal cells of the alloscutal cuticle is a functional feature that contributes to the expansion of the epidermis (and ultimately the cuticle) by changing the cellular configuration from pseudostratified in fasting ticks to single layered in digesting ticks.

As soon as feeding begins, the epidermal cells of the alloscutum become hypertrophic and arrange as a single layered epithelium. We did not find evidence for hyperplasia of epidermal cells in any of the hundreds of sections from numerous ticks we have studied. Lees [2], who studied changes of the epidermis epithelium during the feeding process explicitly, also did not find evidence of hyperplasia. While the lack of evidence is not sufficient to reject a hypothesis, the consistent lack of observed mitotic structures in the epidermis of *Ixodes ricinus* provides some confidence that there is indeed no hyperplasia of epidermal cells. In semi-engorged ticks, the epidermal cells arrange as a prismatic epithelium of large cells (Fig. 3e). While hypertrophy of epidermal cells has been described previously [7, 14], the rearrangement of the cellular configuration of the epithelium is a new detail that adds information for a more complete mechanistic explanation of the area increase of epidermis and cuticle. Once the tick is fully engorged the epidermal cells are fully stretched and are arranged as a thin squamous epithelium (Fig. 3f, g). We feel confident suggesting that an important source of morphological plasticity of the epidermis adjusting to the size changes of the opisthosoma lies in the hypertrophy of epidermal cells and in the change of the arrangement of the cells in the epithelium from a pseudostratified epithelium, to a single layered prismatic epithelium, to a thin squamous epithelium. The occurrence of a pseudostratified epidermal epithelium under the expandable alloscutum, and a simple squamous epidermal epithelium under the non-expandable scutum of females and males, suggests that the pseudostratified epithelium is indeed a functional feature that provides simple and rapid accommodation of size changes of the alloscutum. Indeed, a pseudostratified epithelium is a typical morphological feature to accommodate volume changes of various organ systems (e.g., urinary bladder in vertebrates). The histological results provide evidence that supports our predictions, i.e., the alloscutal epidermis accommodates the size changes of the opisthosoma by hypertrophy of cells, changes of the configuration of the cells in the epithelium, and by changes of the shape of the epithelial cells.

Over the past decades, cellular changes of the digestive cells of the midgut diverticula have attracted considerable interest. The midgut diverticula of fully engorged ticks host a large volume of blood and the epithelium of the midgut diverticula accommodates enormous volume changes. The digestive cells of the epithelium of the midgut diverticula show a tremendous increase in size. In fasting ticks, they are small and more or less cuboidal cells. Already in the semi-engorged ticks digestive cells become large and club-shaped, with their enlarged cytoplasmic part reaching into the lumen of the diverticula. These club-shaped digestive cells change shape during continued engorgement and in fully engorged ticks digestive cells are squamous cells covering the inner side of the diverticula. Our interpretation of these observed differences is that the club-shaped cells of the semi-engorged ticks stretch to accommodate the size increase of the midgut diverticula. Again, changes of the size of cells and the epithelial configuration of these cells can be associated with the overall volume increase of the midgut diverticula. We therefore suggest that size and configuration changes of the digestive cells contribute to the size changes of the midgut diverticula, most certainly in concert with cellular hyperplasia (although this is not proven here). Our random collected material did not allow for a precise timeline of postprandial responses of the midgut diverticula; thus, we cannot provide evidence for cellular hyperplasia that had been described by others [20, 25].

Other studies have reported that digestive cells or parts of them detach from the epithelium and float free in the lumen of the midgut diverticula [10, 20, 30–32]. Agbede and Kemp [20] suggested that those cells may be loaded with indigestible matter and may have excretory functions rather than digestive functions. Agyei et al. [21, 22] suggest that the midgut digestive cells are multifunctional and capable of both secretory and digestive activities; a view that is more recently been supported by Filimonova [23–25] in various mite species. However, Caperucci et al. [33] did not find any detached cells in the lumen of the midgut diverticula of *Amblyomma cajennense* (Acari: Ixodidae).

Theoretically, one could argue that the shape changes of the digestive cells are not based on stretching of the cell body, but on shedding off the apical part and converting a club shape into a squamous shape. We did not find evidence for detaching cells, but cannot exclude with certainty that our random sample missed the relevant postprandial stages. In any case, the two observations would not be mutually exclusive and we suggest that the difference between club-shaped digestive cells in the semi-engorged ticks and squamous cells in fully engorged ticks results from passive stretching of cells during volume increase, but does not exclude the possibility that shedding the apical part of the cells also contributes to the observed changes of cell shape.

Finally, our results provide evidence that the tracheae stretch during the feeding process. In fasting ticks, we find only straight (or slightly bent) tracheae and no evidence of spare material. We reported also light microscopic and ultrastructural details of the wall of the tracheae that are in accordance with our predictions, i.e., volume increase of the idiosoma during feeding results in flattening of the taenidia and increased distance between taenidia. The evidence is strong because we compare fasting and fully engorged animals, thus, compare tracheae in two explicitly different physiological conditions. Based on the analysis of TEM we suggest that the endocuticle of the tracheae provides the elastic properties to stretch during the feeding process while the exocuticle (core of the taenidia) remains relatively unchanged. Expandable tracheae have not been reported before. However, our observations provide a simple mechanistic explanation how the respiratory system adjusts to the enormous volume changes.

## Conclusion

Castor bean ticks are a fascinating example of extreme changes of body volume. While details had been described before, we provide new and additional details that allow for an integrative view considering also configuration changes of epithelial arrangement of cells. Our study provides new insights in how these animals accommodate the enormous volume changes. In particular, changes of epithelial tissue configuration in the epidermis and the midgut diverticula appear to be important components of the morphological response to feeding in ticks. Expandable tracheae have not previously been described in arthropods. The evidence presented here supports this new mechanistic explanation for how tracheae adjust to the enormous size changes of the opisthosoma of ticks.

## Additional files

Additional file 1: **Figure S1.** *Ixodes ricinus,* μCT-Scans of a fasting female. These images document the original surface rendering (red lines) for 3D-reconstructions of tracheae. The entire image stack of 583 images, each labeled for tracheae, is the image basis for the 3D-reconstruction of the tracheal system in text Fig. 6b. (A) Cross section on the level of the spiracular plate and receptaculum seminis. (B) Virtual horizontal section through the same individual. (C) Virtual parasagittal section in a far lateral position through one spiracular opening. Abbreviations: L, walking leg; RS, receptaculum seminis; SP, spiracular plate. (JPG 1532 kb)

Additional file 2: **Figure S2.** *Ixodes ricinus,* light micrographs of histological cross sections through an adult male tick. (A) at the level of the genital opening, (B) level of the genital opening but a little further posterior, (c) section through the posterior region of the idiosoma with two large parts of the anal sac. Abbreviations: AS, anal sac; DVM, dorso-ventral musculature; GO, genital opening; MF marginal fold. (JPG 1169 kb)

Additional file 3: **Figure S3.** *Ixodes ricinus,* light microscopy, high power magnification of a histological sections through the epidermis and cuticle of a semi-engorged female tick. The epidermis forms a single layered epithelium of prismatic cells with centrally located nuclei. Note a relatively large convective trachea floating free in the hemolymphatic space. Abbreviations: DGC, digestive cell; EN, endocuticle; EPD, epidermis; EX, exocuticle; H, hemolymph space; MC, muscle cell; N, nucleus of epidermis cell; T, trachea. (JPG 3281 kb)

Additional file 4: **Figure S4.** *Ixodes ricinus,* light microscopy micrographs of the midgut diverticula of fully engorged females. (A) High power magnification of a light micrograph of a digestive cell, the epidermis and the cuticle. The digestive cells are stretched, now forming a thin squamous epithelium. (B). High power magnification of a light micrograph of a digestive cell, the epidermis and the cuticle. The digestive cells are stretched, now forming a thin squamous epithelium. The nuclei of the digestive cells stain intensively blue with no structuration recognizable (compare to text Fig. 5 for different appearance of nuclei in fasting and semi-engorged ticks). *Abbreviations*: EN, endocuticle; EPD, epidermis; EX, exocuticle; DGC, digestive cell; LMGD, lumen of midgut diverticulum; MC, muscle cell. Black arrowheads in exocuticle point to pore canals. (JPG 2797 kb)
