# Supplementary figures and images for: Morphological responses to feeding in ticks (Ixodes ricinus)
Source: Zoological Lett. 2018 Aug 4;4:20. doi: 10.1186/s40851-018-0104-0 (PMC6091150; doi:10.1186/s40851-018-0104-0)

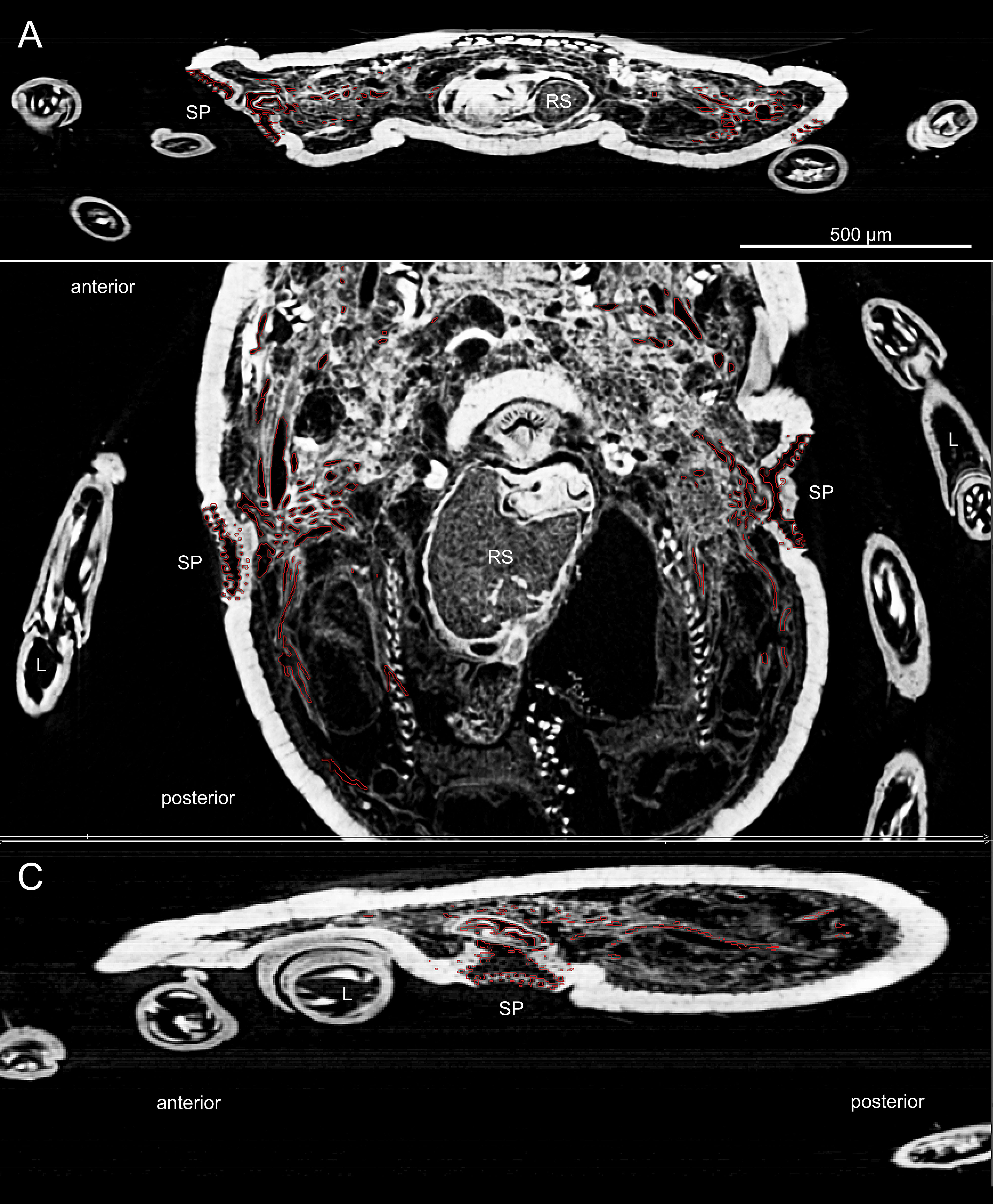

Supplement: Supplementary file 1 — Figure S1. Ixodes ricinus, μCT-Scans of a fasting female. These images document the original surface rendering (red lines) for 3D-reconstructions of tracheae. The entire image stack of 583 images, each labeled for tracheae, is the image basis for the 3D-reconstruction of the tracheal system in text Fig. 6b. (A) Cross section on the level of the spiracular plate and receptaculum seminis. (B) Virtual horizontal section through the same individual. (C) Virtual parasagittal section in a far lateral position through one spiracular opening. Abbreviations: L, walking leg; RS, receptaculum seminis; SP, spiracular plate. (JPG 1532 kb) [file 40851_2018_104_MOESM1_ESM.jpg]

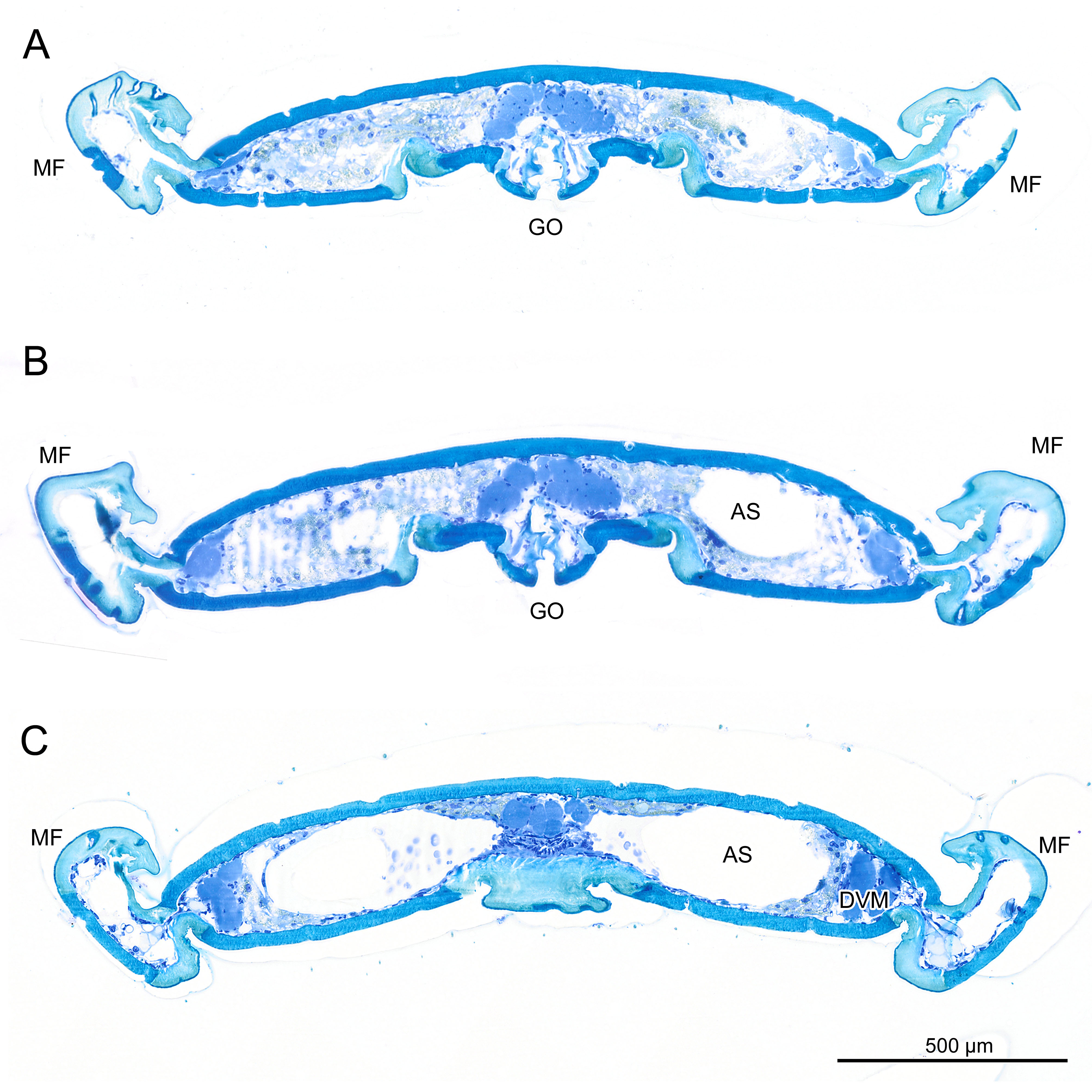

Supplement: Supplementary file 2 — Figure S2. Ixodes ricinus, light micrographs of histological cross sections through an adult male tick. (A) at the level of the genital opening, (B) level of the genital opening but a little further posterior, (c) section through the posterior region of the idiosoma with two large parts of the anal sac. Abbreviations: AS, anal sac; DVM, dorso-ventral musculature; GO, genital opening; MF marginal fold. (JPG 1169 kb) [file 40851_2018_104_MOESM2_ESM.jpg]

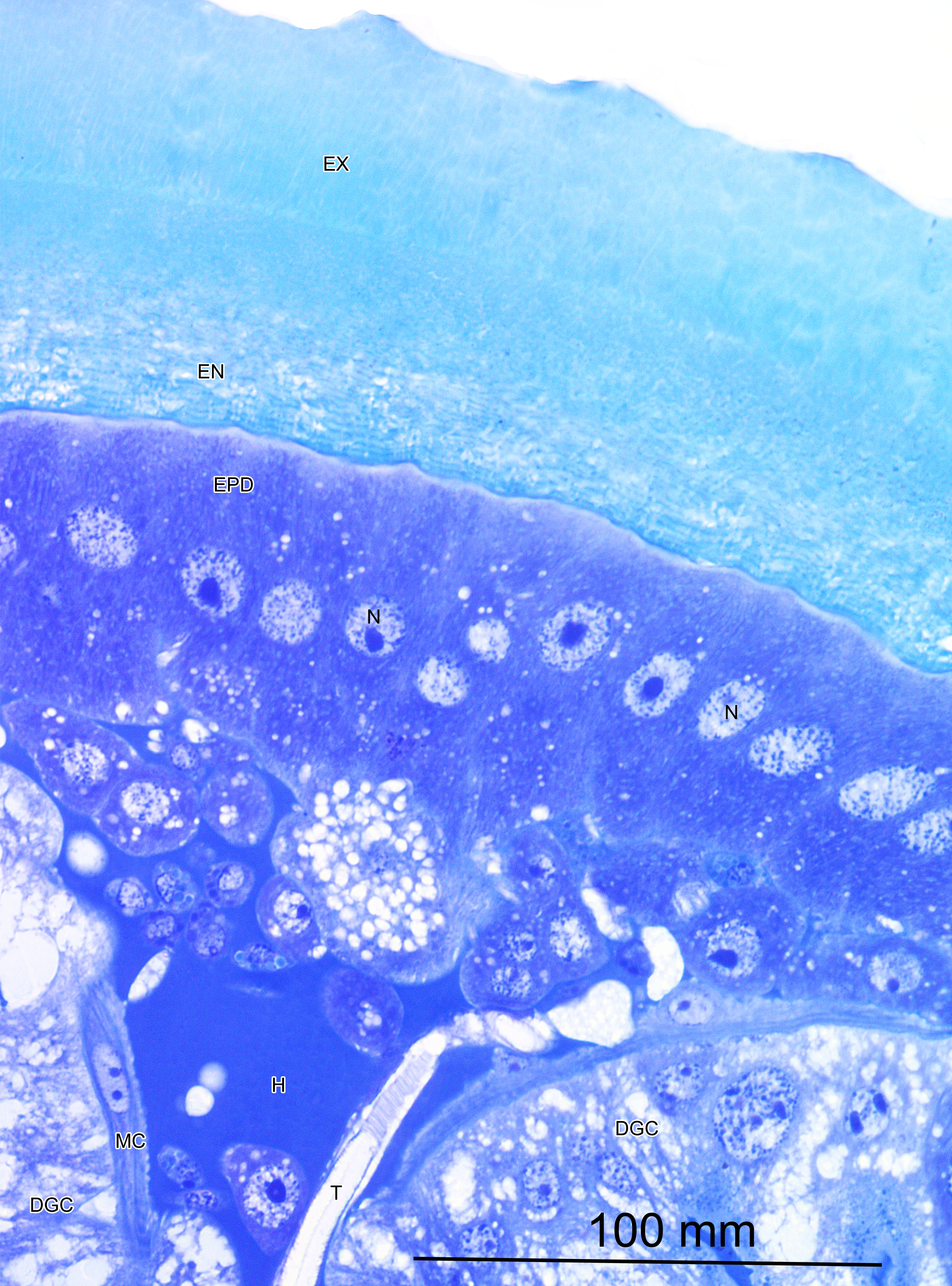

Supplement: Supplementary file 3 — Figure S3. Ixodes ricinus, light microscopy, high power magnification of a histological sections through the epidermis and cuticle of a semi-engorged female tick. The epidermis forms a single layered epithelium of prismatic cells with centrally located nuclei. Note a relatively large convective trachea floating free in the hemolymphatic space. Abbreviations: DGC, digestive cell; EN, endocuticle; EPD, epidermis; EX, exocuticle; H, hemolymph space; MC, muscle cell; N, nucleus of epidermis cell; T, trachea. (JPG 3281 kb) [file 40851_2018_104_MOESM3_ESM.jpg]

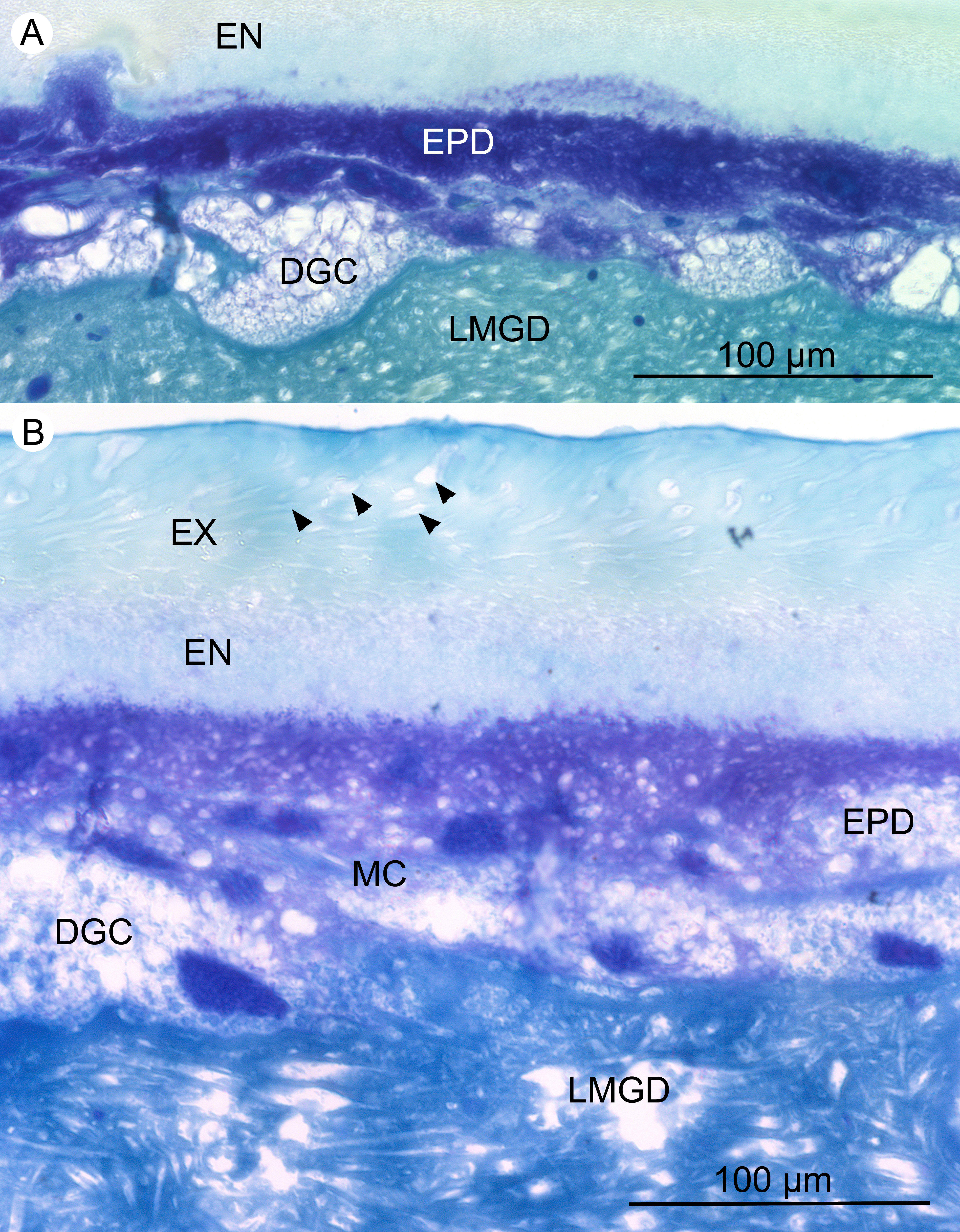

Supplement: Supplementary file 4 — Figure S4. Ixodes ricinus, light microscopy micrographs of the midgut diverticula of fully engorged females. (A) High power magnification of a light micrograph of a digestive cell, the epidermis and the cuticle. The digestive cells are stretched, now forming a thin squamous epithelium. (B). High power magnification of a light micrograph of a digestive cell, the epidermis and the cuticle. The digestive cells are stretched, now forming a thin squamous epithelium. The nuclei of the digestive cells stain intensively blue with no structuration recognizable (compare to text Fig. 5 for different appearance of nuclei in fasting and semi-engorged ticks). Abbreviations: EN, endocuticle; EPD, epidermis; EX, exocuticle; DGC, digestive cell; LMGD, lumen of midgut diverticulum; MC, muscle cell. Black arrowheads in exocuticle point to pore canals. (JPG 2797 kb) [file 40851_2018_104_MOESM4_ESM.jpg]
